# Delayed assessment of serum lactate in sepsis is associated with an increased mortality rate

**DOI:** 10.1186/cc13364

**Published:** 2014-03-17

**Authors:** R Arnold, Z Zhang, S Patel, S Smola, J Isserman, E Jackson

**Affiliations:** 1 Christiana Care Health System, Newark, DE, USA; 2 Cooper University Hospital, Camden, NJ, USA

## Introduction

Lactate assessment early in the resuscitation of sepsis has been recommended as a diagnostic biomarker. An abnormal lactate, independent of blood pressure, is an indication for aggressive fluid resuscitation and its normalization is a recommended endpoint of resuscitation. The objective of this study was to evaluate the effect of the timing of lactate assessment on patient outcomes in sepsis.

## Methods

Data were compiled using the Clinical Vigilance for Sepsis electronic health record (EHR) screening tool, which identified consecutive patients from two hospital systems over 12 months at a 300-bed community hospital and over 24 months from a 500-bed academic tertiary care center. CV Sepsis alert screens the EHR to identify the presence of infection based on a multifactor alert system including labs, vital signs, and treatment team documentation. A physician order for intravenous antibiotics was used as a surrogate for suspected infection. The database identified 37,160 consecutive patients treated for infection from a total of 216,550. Patients with a measured lactate were divided relative to its measurement within 3 hours (eLac) or greater than 3 hours (dLac) of sepsis identification as recommended by the Surviving Sepsis Campaign. The CV Sepsis alert was the reference standard for time zero. Patients were compared in each group for the occurrence of the primary outcome of in-hospital mortality.

## Results

A total 5,072 of 37,160 consecutive patients (13%) had a measured lactate. Sepsis patients experienced an overall 3% (1,186/37,160) mortality rate. In total, 4,153 (82%) patients had measured lactate within 3 hours, and 919 (18%) were delayed, with a decreased morality rate (eLac 6.8 vs. dLac 24.7, *P *< 0.0001). There was no difference in average lactate levels between the groups (eLac: 2.1 ± 2.6, dLac: 2.3 ± 3.0, *P *= NS). A larger ratio of delayed-lactate patients had a lactate ≥4 mmol/l (dLac 12.6% vs. eLac 8.7%).

## Conclusion

The delay in lactate assessment relative to clinical evidence of infection was associated with an increased mortality rate. The average lactate level in each group did not account for this effect. The timing of the assessment, not the lactate level, was prognostic of outcome. The mortality benefit associated with lactate assessment within the 3-hour guideline suggests that an increased clinical awareness may lead to early initiation of time-sensitive interventions known to improve outcomes.

